# Renal atrophy after stereotactic body radiotherapy for renal cell carcinoma

**DOI:** 10.1186/s13014-016-0651-5

**Published:** 2016-05-26

**Authors:** Takaya Yamamoto, Noriyuki Kadoya, Ken Takeda, Haruo Matsushita, Rei Umezawa, Kiyokazu Sato, Masaki Kubozono, Kengo Ito, Yojiro Ishikawa, Maiko Kozumi, Noriyoshi Takahashi, Yu Katagiri, Hiroshi Onishi, Keiichi Jingu

**Affiliations:** Department of Radiation Oncology, Graduate School of Medicine, University of Tohoku, Sendai, Japan; Radiation Technology, Tohoku University Hospital, Sendai, Japan; Department of Radiology, School of Medicine, University of Yamanashi, Yamanashi, Japan

**Keywords:** Stereotactic radiotherapy, SRT, Renal cell cancer, Renal atrophy, Renal remodeling

## Abstract

**Background:**

Renal atrophy is observed in an irradiated kidney. The aim of this study was to determine dose-volume histogram parameters and other factors that predict renal atrophy after 10-fraction stereotactic body radiotherapy (SBRT) for primary renal cell carcinoma (RCC).

**Methods:**

A total of 14 patients (11 males, 3 females) who received SBRT for RCC at Tohoku University Hospital between April 2010 and February 2014 were analyzed. The median serum creatinine level was 1.1 mg/dl and two patients had a single kidney. Nine patients were implanted with fiducial markers. The median tumor diameter was 30 mm. SBRT was delivered at 70 Gy in 10 fractions for 7 tumors, at 60 Gy in 10 fractions for 2 tumors, and at 50 Gy in 10 fractions for 5 tumors with 6 and/or 15 MV X-ray using 5 to 8 multi-static beams. Renal atrophy was assessed using post-SBRT CT images after 12–24 months intervals. Correlations were examined by Spearman rank correlation analysis. Differences between two groups were evaluated by the Mann-Whitney test, and pairwise comparisons were made by the Wilcoxon signed-rank test.

**Results:**

The median tumor volume shrunk from 14.8 cc to 10.6 cc (*p* = 0.12), and the median irradiated kidney volume changed from 160.4 cc to 137.1 cc (*p* < .01). The median peak creatinine level was 1.6 mg/dl after treatment (*p* < .01). Percentage volumes of the irradiated kidney receiving at least 10 Gy (V_10_, *p* = 0.03), V_20_ (*p* < .01), V_30_(*p* < .01), V_40_ (*p* = 0.01), mean irradiated kidney dose (*p* < .01), and magnitude of overlap between PTV and kidney volume (*p* = 0.03) were significantly correlated with post-treatment irradiated kidney volume in percent, and V_20_-V_30_ had strong correlation (*r* < −0.70, *p* < .01). Patients with implanted fiducial markers showed a significantly lower ratio of renal atrophy (*p* = 0.02).

**Conclusions:**

Significant renal atrophic change was observed. Dose distribution of SBRT at 20–30 Gy had a strong correlation with renal atrophy when irradiation was performed in 10 fractions.

## Background

Much progress has been made in extracranial stereotactic radiotherapy since the creation of a stereotactic body frame and application of the frame to treatment [1]. In thoracic malignancies, stereotactic body radiotherapy (SBRT) has become one of the most powerful local therapies and one of the most important treatment options for early stage non-small cell lung cancer, especially in elderly or inoperable patients [2]. Furthermore, pooled analysis of the results of a recent prospective trial have showed that survival rate and regional recurrence rate after SBRT for stage I non-small cell lung cancer were comparable to those after lobectomy [3]. Due to the progress in image-guided radiotherapy, SBRT has been applied to many other sites [4–6]. SBRT has sometimes been used as a definitive treatment for localized prostate cancer [4]. There has been an increasing number of reports on SBRT for hepatocellular carcinoma with a high rate of local control and acceptable toxicities [5].

For renal cell carcinoma (RCC), surgical resection (i.e., nephrectomy) has been the standard treatment. One of the reasons is that RCC is considered to be a radioresistant tumor, but the kidney itself is considered to be relatively radiosensitive [7]. Thus, radiotherapy for RCC has been performed as palliative radiotherapy in most cases or as postoperative radiotherapy in a limited setting [8, 9]. However, in this SBRT era, several outcomes of SBRT for primary RCC have been reported. Although most of the clinical outcomes were reported from single institutions, local control rates were 84–100 % and toxicities rates were relatively low [10–13]. In Japan, a clinical trial has been performed, and our institute took part in the trial starting in April 2010 [14]. This is the multi-center single arm clinical trial of SBRT for RCC, 50 Gy to 70 Gy in 10 fractions were prescribed within dose constraints of the organ at risk and primary endopoints were toxicity and 3-year local progression-free rate.

Because the kidney is a radiosensitive organ, renal atrophy developed after abdominal radiotherapy despite exposures to relatively low doses. A dose-volume histogram (DVH) of renal atrophy 12 months after conventional radiotherapy has been reported [15]. They reported that percentage volumes of the primary irradiated kidney receiving at least 10 Gy, 15 Gy and 20 Gy were significantly associated with renal atrophy. This renal remodeling is also seen 12 months or more after SBRT, but, to the best of our knowledge, there has been only a single case report about post-SBRT renal atrophy [16]. Svedman et al. reported SBRT for 7 renal lesions in patients with one functional kidney [17]. Their results showed that SBRT was safe, and none of their patients required dialysis. Thus, SBRT for RCC is sometimes required for preserving the postoperative remaining kidney. Although SBRT focuses a high dose to a local region with a high level of accuracy, more studies are needed to achieve a higher level of safety. In the present study, DVHs of kidneys treated with a 10-session scheme of SBRT at our institute were retrospectively analyzed. DVH predictive factors for renal atrophy after SBRT intervals of 12–24 months and other factors affecting renal atrophy were investigated.

## Methods

### Patients

Before treatment, all patients with RCC whose maximum tumor diameter was 50 mm or less were assessed by a urologist. For patients who were judged to be inoperable, patients who were judged to be operable but for whom SBRT was preferred and patients who refused surgery, a radiation oncologist assessed the indication for SBRT. SBRT for RCC was performed for 17 consecutive patients at our institute between April 2010 and February 2014. Of those patients, eligibility criteria for current study were availability of follow-up CT images taken 12–24 months after SBRT, no requirement for hemodialysis before SBRT and no additional invasive therapy (such as nephrectomy or radiofrequency ablation) having been performed. Two patients underwent hemodialysis before SBRT and one operable patient declined to continue SBRT and underwent nephrectomy. A total of 14 patients with 14 tumors were retrospectively analyzed. The pretreatment characteristics of the patients are summarized in Table 1. Two patients had previously undergone nephrectomy and therefore had only one kidney. Nine patients were implanted with one to two gold fiducial markers in the kidney parenchyma near the tumor before SBRT. None of the patients had a histologically proven tumor, and assessment by radiologists and a urologist was therefore needed. First, the dynamic CT and MRI were interpreted by two or more radiologists, and agreement with radiographic diagnosis of RCC was needed. Second, the patients underwent examination by a urologist. Operability was judged by the urologist. Assessment that the patient was inoperable, the patient was operable but SBRT was better option than surgical resection, or the patient was operable but refused surgery was needed. Finally, patients received an explanation from a radiation oncologist, and written informed consent was obtained by the radiation oncologist. This study was approved by the Ethical Committee of Tohoku University Hospital (2011-100), and informed consent was obtained from all patients.

**Table 1:** Baseline characteristics of patients

Characteristics	No. (%)
Age, median, y/o	75 (range: 58–87)
Gender	
Female	3 (21)
Male	11 (79)
Creatinine, median, mg/dl	1.1 (range: 0.4–2.0)
Hypertension	
Yes	14 (100)
No	0 (0)
Diabetes mellitus	
Yes	7 (50)
No	7 (50)
Administration of antithrombotic agents	
Yes	10 (71)
No	4 (29)
Past history of nephrectomy	
Yes	2 (14)
No	12 (86)
Fiducial markers	
Yes	9 (64)
No	5 (36)
Tumor diameter, median, mm	30 mm (range: 16–46 mm)

### Treatment protocol and SBRT procedure

Each patient was immobilized in the supine position with a body frame (Vac-loc, Med-tek, Orange City, IA), and movement of the implanted fiducial marker or diaphragm was measured to estimate respiratory tumor motion using continuous X-ray images in a simulator (Ximatron or Acuity system, Varian Medical Systems, Palo Alto, CA). To control respiratory movement, abdominal compression was used in 6 patients and the breath hold technique was used in 1 patient. All patients underwent a planning CT scan at a slice thickness of 2 mm with a multi-detector CT scanner (GE Light Speed Qxi, GE Healthcare, Waukesha, WI); 7 patients underwent a fast CT scan, 5 patients underwent a 4-dimensional CT scan and 2 patients underwent a slow-rotation CT scan (4 s/slice). The internal margin was determined from a planning CT image and from movement of the implanted fiducial marker or diaphragm.

Gross tumor volume (GTV) was defined as visible extent of the tumor on planning CT images, sometimes using CT and MRI fusion images. Clinical target volume (CTV) was equal to GTV. Internal target volume (ITV) was expansion of CTV for the internal margin. A planning target volume (PTV) margin of 5 mm around the ITV was added for patient positioning and set-up uncertainty.

The SBRT plan was created with a 3-dimensional radiotherapy planning system (Eclipse, Varian Medical Systems, Palo Alto, CA), and an analytical anisotropic algorithm (AAA version 8.6.15) was used for dose calculation. Fifty Gy in 10 fractions, 60 Gy in 10 fractions or 70 Gy in 10 fractions covering 95 % of the PTV (D95) was delivered. The prescribed dose was selected on the basis of the highest dose within dose constraints of the organ at risk (Table 2). Seven tumors were prescribed 70 Gy in 10 fractions, 2 tumors were prescribed 60 Gy in 10 fractions and 5 tumors were prescribed 50 Gy in 10 fractions. In only one case, 50 Gy in 10 fractions was delivered to the isocenter to meet dose constraints. The median isocenter dose was 68.8 Gy (range: 50.0–76.4 Gy). SBRT was delivered with a linear accelerator (Clinac 23EX, Varian Medical Systems, Palo Alto, CA) using 6 MV and/or 15 MV X-ray beams with 5 to 8 coplanar and non-coplanar multi-static ports. SBRT was performed on consecutive treatment days. Concomitant tyrosine kinase inhibitor or interferon was not administered.

**Table 2:** Dose constraints for planning organ at risk volume of 10-fraction SBRT

Organ	Constraints	Volume
Irradiated kidney (patient with bilateral kidneys)	30 Gy	Mean
Irradiated kidney (patient with single kidney)	26 Gy	Mean
Lung	20 Gy	≤20 % of total volume
Spinal cord	35 Gy	Any point
Stomach, intestine	52 Gy	≤10 cc
	43 Gy	≤100 cc
Other organs	71 Gy	≤1 cc
	58 Gy	≤10 cc

### Follow-up after SBRT

Patients underwent follow-up examinations every 3 months for 3 years by a radiation oncologist and urologist. CT and MRI were also performed every 6 months for 3 years. When CT examination was performed, enhanced CT images were recommended but were not essential because it was expected that serum creatinine had worsened. Patients often underwent ultrasound examinations of the abdomen, but there were no protocol requirements about ultrasound.

### Renal atrophy assessment

**Fig. 1:**
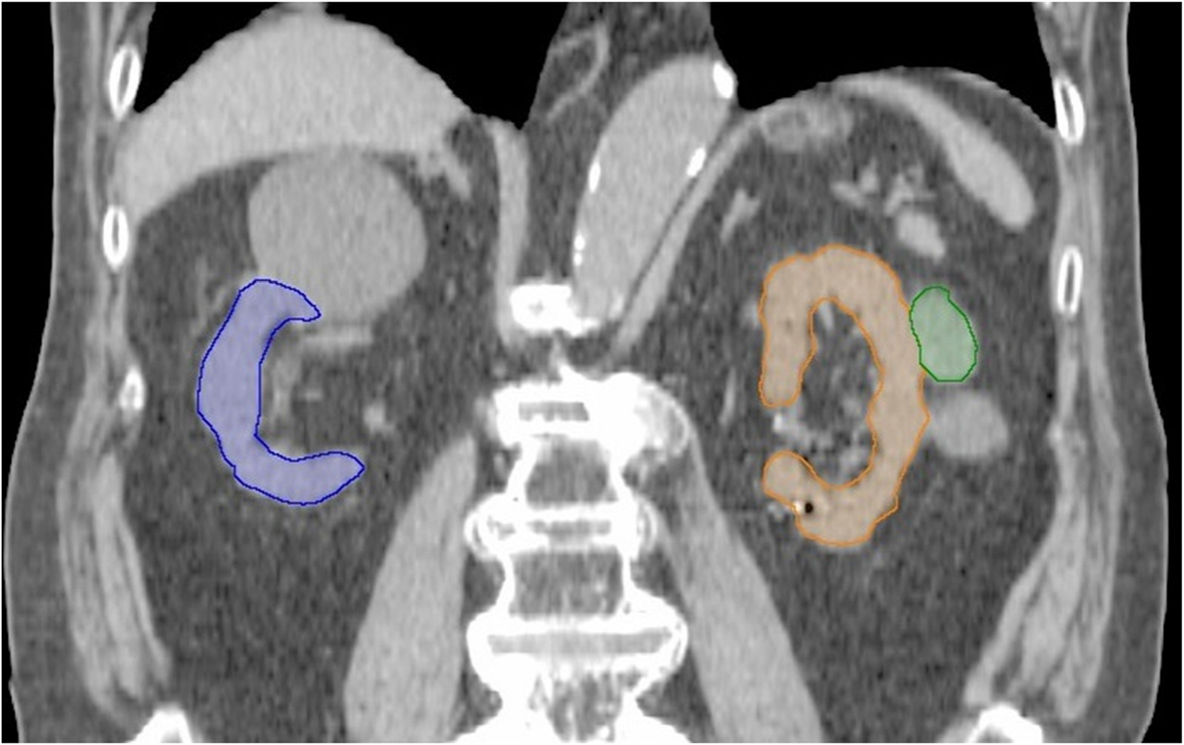
Delineation of functional kidney parenchyma avoiding renal cysts and renal pelvis

Each functional kidney parenchyma was defined as the contoured kidney avoiding renal cysts and renal pelvis (Fig. 1). Kidney volume was defined as functional kidney parenchyma minus GTV. Pretreatment functional kidney parenchyma was delineated using planning CT images. When the slow-rotation scanning technique was used for radiotherapy planning, the use of pretreatment diagnostic CT and planning CT rigid fusion images were permitted to avoid overestimation of kidney volume. When the non-contrast enhanced CT images were used for radiotherapy planning, the use of diagnostic MRI and planning CT rigid fusion images were permitted because there was no difference between CT and MRI for volumetry [18]. Post-SBRT kidney volume was assessed using follow-up diagnostic CT images with a slice thickness ≤ 2 mm, and the use of follow-up CT and follow-up MRI fusion images was permitted as appropriate. Volumetric analysis was performed using Eclipse. The DVH parameter regarding kidney volume was analyzed using V_n_ (%), which was defined as percentage volume of the kidney receiving at least n dose in Gy. Percent changes in parameters was defined as post-SBRT parameters divided by pre-SBRT parameters in percent.

### Statistical analysis

Time to an event was calculated from the first day of SBRT to the day an event was confirmed. Toxicity was graded according to the National Cancer Institute Common Terminology Criteria for Adverse Events version 4.0. Correlations between continuous variables were examined by Spearman’s rank correlation, and *r* was the correlation coefficient. Simple linear regression was applied to create a linear regression equation. Differences in continuous variables between two groups were evaluated by the Mann-Whitney test. Changes from pre-SBRT parameters to post-SBRT parameters were assessed using the Wilcoxon signed-rank test. A *p*-value < .05 was defined as significant in all tests. JMP Pro v.11.2 (SAS Institute, Cary, NA) was used for statistical analyses.

## Results

### Toxicity and parameter changes

**Fig. 2:**
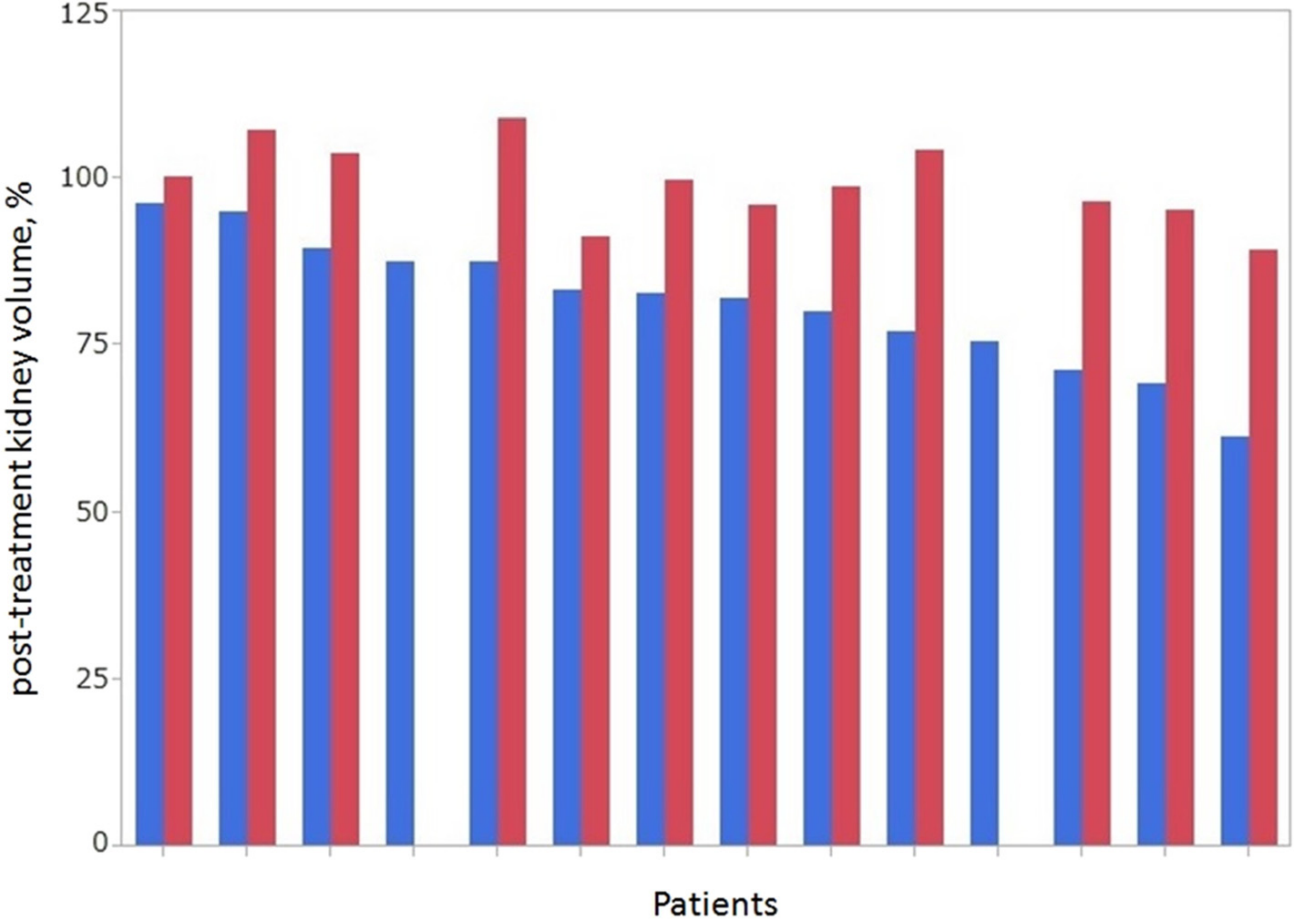
Each median percent change in irradiated kidney volume (*blue*) and contralateral kidney volume (*red*)

**Fig. 3:**
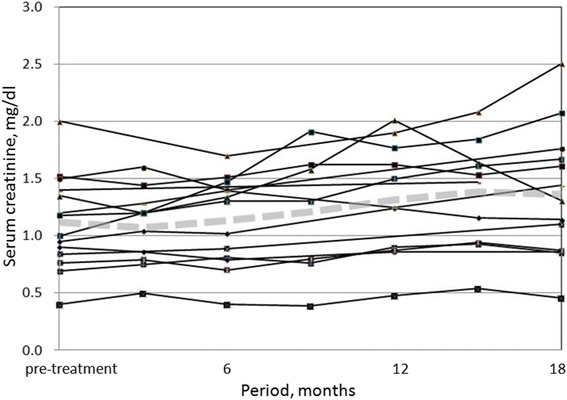
Serum creatinine levels over time. Figure 3 shows changes in serum creatinine levels of each patient over time, and the *grey dashed line* shows mean serum creatinine over time

The median interval between SBRT and renal atrophy assessment CT was 16.9 months (range: 12.0–21.8 months), and no patient was administered tyrosine kinase inhibitor or interferon during that interval. The median tumor volume shrunk from 14.8 cc (range: 3.0–55.6 cc) to 10.6 cc (range: 1.3–38.9 cc, *p* = 0.12). The median post-treatment tumor volume in percent was 73.4 % (range: 41.5–144.6 %). Change in median irradiated kidney volume was from 160.4 cc (range: 99.4–295.5 cc) to 137.1 cc (range: 70.6–258.7 cc, *p* < .01). The median post-treatment irradiated kidney volume in percent was 82.6 % (range: 61.3–96.4 %, Fig. 2). Change in median contralateral kidney volume was from 147.0 cc (range: 118.1–183.6 cc) to 143.9 cc (range: 114.5–191.8 cc, *p* = 0.73). The median post-treatment contralateral kidney volume in percent was 99.3 % (range: 89.4–109.0 %, Fig. 2). The median follow-up period for all patients was 31.2 months (range: 16.2–54.2 months). During follow-up, no grade 2 or higher renal and gastrointestinal toxicity occurred and there was no intervention. None of the patients had progression of hypertension and none of the patients required hemodialysis. The pre-SBRT median serum creatinine level was 1.1 mg/dl (range: 0.4–2.0 mg/dl). The post-SBRT peak value seen in intervals of 0.3–48.1 months was 1.6 mg/dl (*p* < .01), and the post-SBRT value at the time of reporting was 1.3 mg/dl (*p* = 0.05). Serum creatinine of each patient over time was showed in Fig. 3.

#### Univariate analysis

Spearman’s correlations between post-treatment irradiated kidney volume in percent and continuous variables are shown in Table 3, and the results of Mann-Whitney test for differences between post-treatment irradiated kidney volume in percent and categorical variables are shown in Table 4. Significant correlation were seen for V_10_ (*r* = −0.56, *p* = 0.03), V_15_ (*r* = −0.68, *p* < .01),V_20_ (*r* = −0.76, *p* < .01), V_30_ (*r* = −0.71, *p* < .01), V_40_ (*r* = −0.65, *p* < .01), mean irradiated kidney dose (*r* = −0.66, *p* < .01), and magnitude of overlap between PTV and kidney volume (*r* = −0.56, *p* = 0.03), on the other hand, PTV did not have a correlation (*r* = −0.03, *p* = 0.91). The observed factor of post-treatment contralateral kidney volume in percent also had correlation (*r* = 0.65, *p* = 0.02). There was a significant difference between the post-treatment irradiated kidney volume in percent with and without fiducial markers. In patients with fiducial markers and those without fiducial markers, the post-treatment irradiated kidney volumes in percent were 87.5 % (range: 75.6–96.4 %) and 71.4 % (range: 61.3–83.4 %, *p* = 0.02), respectively, but the median isocenter doses were 73.4 Gy (range: 50.0–76.4 Gy) and 65.5 Gy (range: 53.7–73.6 Gy), respectively, and the isocenter doses were not significantly different (*p* = 0.54). Further detailed investigation using Spearman’s correlations revealed that V_24_ showed the strongest correlation (*r* = −0.778, *p* < .01). A simple linear regression for the post-treatment irradiated kidney volume in percent and V_24_ was used for predicting a linear equation. As a result, the following linear regression equation was obtained:

**Table 3:** Spearman’s correlations between post-treatment irradiated kidney volume in percent and continuous variables

	Parameters		Correlation of irradiated kidney	
Variables	Median	Range	Correlation coefficient	*P* value
V_5_ (%)	73.7	43.3–91.8	−0.44	0.11
V_10_ (%)	67.3	34.7–82.5	−0.56	0.03*
V_15_ (%)	62.5	28.8–67.9	−0.68	< .01*
V_20_ (%)	53.5	24.1–64.6	−0.76	< .01*
V_25_ (%)	46.8	20.8–61.1	−0.77	< .01*
V_30_ (%)	41.9	18.3–55.4	−0.71	< .01*
V_40_ (%)	33.8	14.5–47.5	−0.65	0.01*
V_50_ (%)	20.7	6.3–42.1	−0.43	0.12
V_60_ (%)	13.6	0–35.5	−0.19	0.49
Mean irradiated kidney dose (Gy)	27.5	12.9–37.6	−0.66	< .01*
Pre-SBRT irradiated renal volume (cc)	160.4	99.4–295.5	0.07	0.79
GTV (cc)	14.8	3.0–55.6	0.39	0.16
PTV (cc)	79.8	28.1–146.9	−0.03	0.91
Magnitude of overlap between PTV and kidney volume (cc)	19.8	7.9–50.3	−0.56	0.03*
post-treatment contralateral kidney volume in percent (%, *n* = 12)	99.3	89.4–109.0	0.65	0.02*
Isocenter dose (Gy)	68.8	50.0–76.4	0.07	0.80
Age (years)	75	58–87	0.03	0.89
Creatinine change ratio (peak value/pre-SBRT) (%)	139.5	105.7–190.0	0.32	0.26

Abbreviations: *V*_*n*_ percentage volume of the irradiated kidney receiving at least n dose in Gy, *p < .05

**Table 4:** Mann-Whitney test between post-treatment irradiated kidney volume in percent and categorical variables

	Change ratio of subgroup 1		Change ratio of subgroup 2		Difference
Variables	Median	Range	Median	Range	*P* value
Gender (male, female)	82.8	69.4–96.4	75.6	613–87.5	0.31
Diabetes mellitus (Yes, No)	83.4	61.3–96.4	82.1	69.4–89.5	0.65
Antithrombotic drug (Yes, No)	82.5	69.4–96.4	81.6	61.3–95.0	0.88
Fiducial markers (Yes, No)	87.5	75.6–96.4	71.4	61.3–83.4	0.02*
Abdominal compression	81.7	69.4–96.4	82.5	61.3–95.0	0.74
4-dimensional CT (Yes, No)	82.2	61.3–96.4	82.5	71.4–89.5	1.00

*p < .05

$$ \mathrm{Estimated}\kern0.5em \mathrm{post}\hbox{-} \mathrm{treatment}\ \mathrm{irradiated}\ \mathrm{kidney}\ \mathrm{volume}\left(\%\right) = 111.4\ \hbox{-}\ 0.659\ *\ {\mathrm{V}}_{24}\left(\%\right) + \upvarepsilon $$

**Fig. 4:**
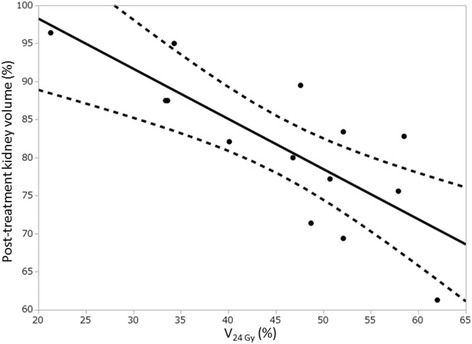
Scatter plot and estimated linear regression equation. Scatter plot and estimated regression equation as a result of linear regression analysis of post-treatment irradiated kidney volume in percent with V_24_ are shown. *Dashed lines* represent 95 % confidence bands. The simple linear regression equation is as follows: Estimated post-treatment irradiated kidney volume (%) = 111.4**–**0.659 * V_24_ (%) + ε. Epsilon is the error term and its standard deviation is 6.43. The coefficient of determination was 0.60 (*p* < .01)

Epsilon is the error term and its standard deviation is 6.43. The coefficient of determination was 0.60 (*p* < .01, Fig. 4).

Analysis for the contralateral kidney was also performed (*n* = 12). The DVH for the contralateral kidney showed that median V_5_ was 0.0 % (range: 0.0–16.0 %), V_10_ was 0.0 % (range: 0.0–0.4 %), and median value of mean contralateral kidney dose was 1.1 Gy. Post-treatment contralateral kidney volume in percent was 99.3 % (range: 89.4–109.0 %). Contralateral kidneys were almost unexposed to radiation, and renal atrophy was not seen. Spearman’s correlation and Mann-Whitney test were also applied, but no significant factor for post-treatment contralateral kidney volume in percent emerged (data not shown).

## Discussion

This study was one of the few studies in which kidney DVH parameters and other factors were analysed to find predictors of renal atrophy after SBRT and to identify the correlation between post-treatment irradiated kidney volume in percent and kidney V_n_. The results showed that V_20_-V_30_ had strong correlation (*r* < −0.70, *p* < .01) and that V_24_ had the strongest correlation with renal atrophic change, and a linear equation of estimated post-treatment irradiated kidney volume in percent with V_24_ was obtained (Fig. 4). V_10_-V_40_, mean irradiated kidney dose, magnitude of overlap between PTV and kidney volume, and post-treatment contralateral kidney volume in percent had significant relationships with renal atrophy. Patients with implanted fiducial markers showed a significantly lower ratio of renal atrophic change. Compensatory hypertrophy of the contralateral kidney was not observed.

The correlation between V_20_ to V_30_ and renal atrophy was compatible with previous findings, but the values were slightly higher than previous findings considering the number of fractionations [15]. There were some differences from previous reports. First, because SBRT focuses a high dose to a local region with precise respiratory motion management, kidney DVH was less affected by interfractional and intrafractional kidney movement than that in conventional fractionation series. Owing to the converged dose distribution, the area outside the PTV and the contralateral kidney were minimally irradiated. Second, no concurrent chemotherapy or adjuvant chemotherapy was used in this study. Third, there were some differences in morphological assessment, such as the measurement of craniocaudal length on CT images [15]. Finally, the analysis in this study was not analysis of bilateral kidneys but analysis of each kidney, and compensatory hypertrophy of the contralateral kidney therefore had less effect on the analysis (but compensatory hypertrophy was not seen in this study) [19]. For either reason, attention must be given to V_20_ to V_30_ of the kidney in a 10-session scheme of SBRT, but, unfortunately, a clinically meaningful cut-off value was vague in this study because of the limited sample size and various comorbidities.

Although the post-treatment irradiated kidney volume in percent was not correlated with serum creatinine, some surgical series for solitary kidney patients have shown a relationship between renal function and residual kidney volume or volume in percent [18, 20, 21]. Sharma et al. reported that the correlation between percent change in kidney parenchymal volume and percent change in GFR (pre-/postoperative GFR) was moderate but statistically significant. In the present study, only two patients with a solitary kidney were enrolled, and further analysis could therefore not be performed. In a unilateral kidney situation, spared kidney volume from V_n Gy_ may also be important considering surgical results and radiation pneumonitis analyses [18, 22].

The post-treatment irradiated kidney volume in percent also had a moderate positive correlation with the post-treatment contralateral kidney volume in percent though this factor was not predicting factor but observed one (*r* = 0.65, *p* = 0.02). The post-treatment contralateral kidney volume in percent was 99.3 %, resulting in no occurrence of the compensatory hypertrophy of contralateral kidney, but that ratio ranged from 89.4 to 109.0 % and had a significant correlation with the post-treatment irradiated kidney volume in percent. This result, that is, a tendency for enlargement of the contralateral kidney with lower post-treatment irradiated kidney volume in percent, suggested that host factors could affect renal remodeling even in a setting with no additional treatment. Patients with little reserved kidney capacity would show more atrophic change because of the irradiation and host factors, and the converse might also occur. Host factors in this study included age and comorbidities such as cardiovascular disease (all of the patients in this study needed administration of an antihypertensive agent and 71 % of the patients needed administration of antithrombotic).

A lower ratio of post-treatment kidney volume was seen in patients with implanted fiducial markers (*p* = 0.02), though prescribed doses were not different (*p* = 0.54). These results were thought to be partly caused by the contribution of fiducial markers to reduction of the internal and set-up margins. In patients with fiducial markers and those without fiducial markers, the mean values of PTV were 84.0 and 83.8 cc, respectively (*p* = 0.99), and the mean values of internal volume and set-up volume (i.e., PTV minus GTV) were 64.0 and 72.8 cc, respectively (*p* = 0.59). In case of RCC, it is often difficult to distinguish the tumor from kidney parenchyma on non-enhanced CT images and even more difficult on non-enhanced cone beam CT images. On the other hand, fiducial markers were easy to identify on non-enhanced cone beam CT images, and the markers allowed us to confirm interfractional and intrafractional motions by using continuous X-ray images of a linear accelerator (on-board imager; Varian Medical Systems, Palo Alto, CA). These facts have probably made radiation oncologists and medical physicists extend the internal and set-up margins when gold fiducial markers were not implanted. Although PTV did not have a relationship with post-treatment irradiated kidney volume in percent, the magnitude of overlap between PTV and kidney volume had a moderate but significant relationship (Table 4). This indicated that not only the internal margin but also location of the tumor in the kidney were important factors for renal atrophy. When the magnitude of overlap between PTV and kidney volume is expected to increase because of the tumor location, implantation of fiducial markers is recommended to reduce the internal margin and renal atrophy.

There are several limitations in the current study. This study was a study conducted in a single institute with a limited sample size, and patient comorbidities were not controlled. Therefore, the exact cut-off value was vague and a clinically meaningful cut-off value was not obtained. The radiotherapy planning CT methods varied, and this affected volumetric analyses to some extent. Studies with a larger sample size and further analyses including multivariate analyses were needed. The periods of assessment CT ranged from 12.0 months to 21.8 months after SBRT. A smaller distribution of SBRT and assessment CT intervals would enable more precise renal atrophy analysis.

## Conclusions

V_10_ to V_40_ of the irradiated kidney and mean irradiated kidney dose had significant correlations with the post-treatment irradiated kidney volume in percent, with V_20_ to V_30_ showing strong correlations after SBRT for primary RCC. Attention must be paid to the dose distribution at 20 Gy to 30 Gy in 10 fractions in SBRT for RCC. Implantation of fiducial markers might be beneficial to reduce renal atrophy by reducing the internal margin.

## Abbreviations

AAA: analytical anisotropic algorithm
CTV: clinical target volume
DVH: dose-volume histogram
GTV: gross tumor volume
ITV: internal target volume
PTV: planning target volume
RCC: renal cell carcinoma
SBRT: stereotactic body radiotherapy
